# Exploring the role of survivin in neuroendocrine neoplasms

**DOI:** 10.18632/oncotarget.27631

**Published:** 2020-06-09

**Authors:** Ahmad Hanif, Sunyoung Lee, Medhavi Gupta, Ankush Chander, Eric D. Kannisto, Achamaporn Punnanitinont, Robert Fenstermaker, Michael Ciesielski, Kristopher Attwood, Jingxin Qiu, Sai Yendamuri, Renuka Iyer

**Affiliations:** ^1^ Department of Medicine, Roswell Park Comprehensive Cancer Center, Buffalo, NY 14263, USA; ^2^ Department of Pathology, Roswell Park Comprehensive Cancer Center, Buffalo, NY 14263, USA; ^3^ Department of Biostatistics and Bioinformatics, Roswell Park Comprehensive Cancer Center, Buffalo, NY 14263, USA; ^4^ Department of Thoracic Surgery, Roswell Park Comprehensive Cancer Center, Buffalo, NY 14263, USA; ^5^ Department of Neurosurgery, Roswell Park Comprehensive Cancer Center, Buffalo, NY 14263, USA

**Keywords:** neuroendocrine tumors, survivin, immunohistochemistry, biomarkers, radiosensitivity

## Abstract

Neuroendocrine tumors (NETs) are a heterogenous group of tumors. While most NETs have excellent prognosis, certain subsets have aggressive biology and have limited treatment options. We explored the role of survivin in NET as a prognostic and potentially therapeutic marker. Tissue microarrays of 132 patients were stained for survivin using immunohistochemistry (IHC) and correlated with outcomes. Using genomic database, we then correlated survivin (BIRC5) mRNA expression with radiosensitivity index (RSI) in 52 samples of NET. Finally, we studied the effect of radiation on survivin expression in human cell lines and the impact of knock-down of BIRC5 on cell proliferation and radiation sensitivity. We found that survivin positivity by IHC correlated with a shorter survival (overall survival 8.5 years vs. 18.3 years, *p* < 0.001). There was a positive correlation between BIRC5 expression and RSI (r = 0.234, *p* < 0.0001). Radiation exposure increased BIRC5 gene expression in a human carcinoid cell line. Knockout of BIRC5 using siRNA reduced proliferation of neuroendocrine cells but did not increase radiation sensitivity. We conclude that survivin expression in NET correlates with an inferior survival and survivin expression in human carcinoid cell lines increases after exposure to ionizing radiation.

## INTRODUCTION

Neuroendocrine tumors (NET) are a heterogeneous group of neoplasms that arise from neuroendocrine cells or their precursors. They are considered rare cancers, but the incidence has increased over the last 30 years to 6.98 cases per 100,000 population per year [1, 2]. NET can occur throughout the body but are mostly associated with the digestive or bronchopulmonary systems. Classification of NET range from well-differentiated neuroendocrine tumors to poorly differentiated neuroendocrine carcinomas (NEC) based on their morphology and histological grade assessed by Ki-67 proliferation index or number of mitoses per 10 high-powered fields [3]. Lung NETs can additionally be classified as low grade, typical and atypical carcinoids, or high-grade carcinomas, small or large cell [4].

Most NET are indolent and associated with excellent prognosis. However, metastatic NET represent aggressive disease and median overall survival (OS) for metastatic pancreatic and small bowel NET is 24 and 56 months, respectively [1]. Lung NET are generally more aggressive and are associated with a worse overall survival of around 17 months. The standard of care for metastatic NET is somatostatin analogues (well differentiated NET) or chemotherapy (poorly differentiated NEC) [5]. Tumors that progress on first-line therapies have limited systemic options that include cytotoxic chemotherapy, molecularly targeted therapy, interferon-α and more recently, peptide receptor radioligand therapy (PRRT). However, responses to second-line therapies are generally short lived (~1 year) and many patients are unable to tolerate associated toxicities. Thus, there is an urgent need for new therapies for metastatic NETs. Novel immunotherapy and biomarker selected studies / rational combinations are needed.

Survivin is a 16.5 kDa intracellular protein that belongs to the inhibitor of apoptosis protein (IAP) family. It interacts with mitotic spindle apparatus to regulate cell division and has also been shown to modulate the function of a number of effector cell death proteases (caspase-3 and caspase-7) leading to inhibition of apoptosis [6, 7]. It is ubiquitously expressed in embryonic and fetal tissue but its expression in normal tissues of adult humans is limited to hematopoietic progenitor cells, some lymphocytes, neutrophils and vascular endothelial cells [8]. Survivin is over-expressed in most cancers and its expression has been correlated with development of resistance to anti-cancer therapy in pre-clinical studies [9–11]. Based on these observations, survivin has recently emerged as an attractive target for resistant malignancies that lack effective therapies.

To test the potential of survivin as a prognostic marker and therapeutic target we evaluated survivin expression in NET and correlated it with clinical outcomes using annotated tumor tissue microarrays from patients with NETs. Lung NETs have the fewest therapy options and are under-represented in clinical trials due to the relatively rare incidence. To better understand the role if any of survivin in NETs of lung origin, using a genomic database we correlated the survivin mRNA expression with sensitivity to radiation using the validated radio-sensitivity index (RSI). Finally, to determine if survivin targeting may increase response to radiation, we assessed change in survivin expression in a pulmonary carcinoid cell line in response to radiation and effects of survivin knockdown using siRNA on cell proliferation and radiation sensitivity. Our goal is to use this data to build more effective therapies for NET and provide rational support for new trials for these patients with high unmet need.

## RESULTS

### Survivin expression in NET tissue microarrays (TMAs)

**Figure 1:**
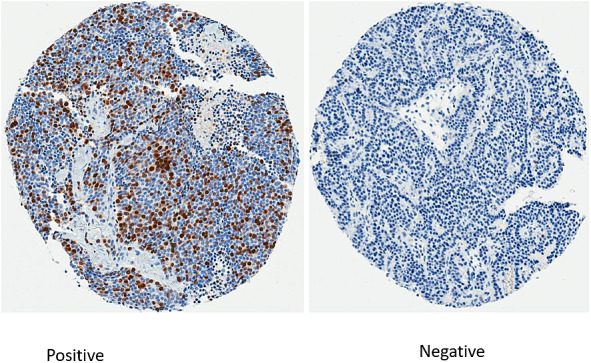
Immunohistochemistry staining for survivin.

Of 167 tumor samples in the TMAs, 132 were of good quality and analyzable for survivin expression by IHC. Out of these, 68 (52%) were survivin positive and 64 (48%) were negative. The immunohistochemistry (IHC) staining for survivin is shown is Figure 1. Baseline characteristics of patients by survivin expression on tumor are summarized in Table 1. Significant associations between survivin expression and age, smoking status, primary site, grade and tumor size were seen. Median age was higher in survivin positive group (60.5 years vs 54 years, *p* = 0.004). However, when patients were divided based on age cut-off of 60 years, survivin negative group had a greater number of patients older than 60 years (71.9% vs 47.1%, *p =* 0.005). Forty (59%) out of 68 survivin positive tumors were lung NET followed by 16 (24%) from gastro-enteropancreatic (GEP) origin. Patients with survivin positive tumors were more likely to be smokers; 57 (83.8%) of patients in survivin positive had tobacco exposure, either active or past, compared to 32 (50%) in survivin negative group, *p <* 0.001. Survivin positive tumors tended to be larger, mean tumor size was about 6 cm larger at the time of diagnosis. There were no significant associations between survivin expression and sex, race, stage at diagnosis. Rates of upfront surgery were similar in survivin positive and negative groups, with 94.1% and 98.4% patients undergoing surgical resection in each group respectively.

**Table 1:** Baseline characteristics of patients by survivin expression on tumor

		Negative (Survivin Score 0%)	Positive (Survivin Score >0%)	Overall	*P*-value
Overall	*N*	64 (48.5)	68 (51.5)	132 (100%)	
Age, y	Median (Range)	54.0 (21–82)	60.5 (27–89)	58.0 (21–89)	0.004
Age by group	< 60 years	18 (28.1%)	36 (52.9%)	54 (40.9%)	0.005
	> 60 years	46 (71.9%)	32 (47.1%)	78 (59.1%)	
Sex	Male	21 (32.8%)	26 (38.2%)	47 (35.6%)	0.59
	Female	43 (67.2%)	42 (61.8%)	85 (64.4%)	
Race	White	58 (90.6%)	64 (94.1%)	122 (92.4%)	0.68
	Black	4 (6.3%)	3 (4.4%)	7 (5.3%)	
	Other	2 (3.1%)	1 (1.5%)	3 (2.3%)	
Smoking Status	Never	32 (50.0%)	11 (16.2%)	43 (32.6%)	<0.001
	Former	18 (28.1%)	30 (44.1%)	48 (36.4%)	
	Active	14 (21.9%)	27 (39.7%)	41 (31.1%)	
Primary Site	Lung	22 (34.4%)	40 (58.8%)	62 (47.0%)	0.003
	Pancreas	14 (21.9%)	5 (7.4%)	19 (14.4%)	
	Small Intestine	18 (28.1%)	11 (16.2%)	29 (22.0%)	
	Other	7 (10.9%)	12 (17.6%)	19 (14.4%)	
	Unknown	3 (4.7%)		3 (2.3%)	
Grade	I	36 (61.0%)	17 (26.2%)	53 (42.7%)	<0.001
	II	12 (20.3%)	10 (15.4%)	22 (17.7%)	
	III	11 (18.6%)	38 (58.5%)	49 (39.5%)	
CgA	Negative	5 (11.4%)	13 (26.5%)	18 (19.4%)	0.07
	Positive	39 (88.6%)	36 (73.5%)	75 (80.6%)	
Stage	1	21 (33.3%)	27 (40.3%)	48 (36.9%)	0.45
	2	12 (19.0%)	11 (16.4%)	23 (17.7%)	
	3	11 (17.5%)	16 (23.9%)	27 (20.8%)	
	4	19 (30.2%)	13 (19.4%)	32 (24.6%)	
Tumor Size (cm)	Median (Range)	20.0 (0.1–150)	27.0 (8.0–100)	25.0 (0.1–150)	0.003
Tumor Size by group	≤15 cm	17 (29.3%)	5 (7.6%)	22 (17.7%)	0.003
	16–40 cm	10 (17.2%)	22 (33.3%)	32 (25.8%)	
	> 40 cm	31 (53.4%)	39 (59.1%)	70 (56.5%)	
TPH expression	Negative (≤ 1)	11 (18.3%)	20 (30.3%)	31 (24.6%)	0.15
	Positive (> 1)	49 (81.7%)	46 (69.7%)	95 (75.4%)	
Ki-67 Grade	Low (<3%)	59 (92.2%)	47 (72.3%)	106 (85.5%)	<.001
	High (≥3%)	5 (7.8%)	18 (27.7%)	18 (14.5%)	

### Survivin expression in NET patients is associated with aggressive disease

In terms of histology, survivin expression was associated with higher grade and high Ki-67 index. All tumor specimens lacking survivin expression were in the low Ki-67 group. When divided by grade, only 17 (26.2%) of tumors in survivin positive group were low grade compared to 36 (61%) in survivin negative group, *p <* 0.001 (Table 1). Chromogranin A expression was reported in 93 patients and there was no correlation between expression of chromogranin A on tumor surface and survivin positivity. Since high urine 5-hydroxyindoleaceticacid (5-HIAA) is a poor prognostic factor in NETs [12, 13], we used tryptophan hydroxylase (TPH) staining as a surrogate marker to evaluate for any differences in TPH staining between the two groups which can impact prognosis [14]. The number of samples expressing TPH was not significantly different between the two groups and there was no correlation between survivin and TPH expression (Spearman’s correlation coefficient, *r*_s_ = -0.17, *p* = 0.06). Overall, patients with survivin positive tumors were more likely to be older, with larger, high grade tumors, and have tobacco exposure (Table 1).

### Survivin expression in NET patients predicts a shorter survival

**Figure 2:**
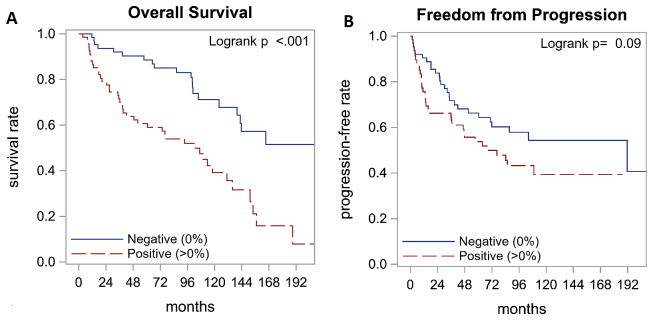
Survival outcomes by survivin expression. Analysis of survival outcomes with immunohistochemistry staining for survivin indicated that overall survival is better in patients with survivin negative tumors (**A**). Freedom from progression was not significantly different between the two groups (**B**).

Survival outcomes by survivin expression are summarized in Table 2. After a median follow up of 9.8 years, survivin positivity was found to be associated with an inferior median overall survival (8.5 years vs 18.3 years, *p <* 0.001) with hazard ratio (HR) of 2.89 (95% CI: 1.68-4.95; Figure 2A). There was a trend towards worse freedom from progression (FFP) after first line therapy in survivin positive patients (5.6 years vs 15.9 years, *p* = 0.09) with HR of 1.55 (95% CI: 0.93-2.59) that was not statistically significant (Figure 2B). However, in the context of high variability in first line, this non-statistical difference in FFP carries limited prognostic significance.

**Table 2:** Survival outcomes by survivin expression

		5-yr Rate, % (95% CI)	10-yr Rate, % (95% CI)	Median Time, months (95% CI)	Median Follow-up, months (Range)
Overall Survival	Total	74 (65–81)	54 (44–64)	135.9 (106.8–156.9)	118.1 (0.9–230.6)
	Negative (0%)	89 (77–94)	71 (56–82)	220.1 (139.8–NR)	121.3 (3.4–230.6)
	Positive (>0%)	61 (48–71)	39 (26–52)	102.9 (48.7–130.8)	115.7 (0.9–209.5)
Freedom from progression	Total	60 (51–68)	47 (37–56)	104.4 (60.5–NR)	
	Negative (0%)	66 (53–77)	54 (39–67)	191.5 (70.6–NR)	
	Positive (>0%)	54 (40–65)	39 (26–53)	67.7 (36.3–NR)	

**Figure 3:**
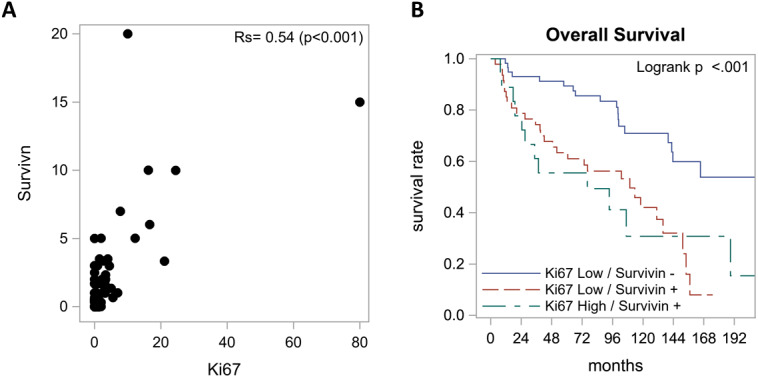
Relationship between survivin expression and Ki-67 index. (**A**) Spearman correlation indicated a moderately positive correlation between survivin expression and Ki-67 index. (**B**) Overall survival according to survivin and Ki-67. Best survival was seen in Ki-67 Low/survivin negative group with a median OS of 18.3 years followed by Ki-67 Low/survivin positive group with 9.1 years and Ki-67 High/ survivin positive group with 6.3 years.

We found a moderate positive correlation between survivin expression and Ki-67 index where survivin positive tumors tended to have high Ki-67 index (*r*_s_ = 0.54, *p <* 0.001, Figure 3A). We also performed exploratory analysis of survivin with Ki-67 which led to the formation of three distinct groups with respect to overall survival (Table 3). Patients with Ki-67 Low/survivin negative tumors had the best outcomes with median overall survival of 18.3 years followed by Ki-67 Low/survivin positive tumors with 9.1 years and Ki-67 High/survivin positive tumors with 6.3 years (*p <* 0.001). See Figure 3B for survival curves with respect to Ki-67 index and survivin expression.

**Table 3:** Overall survival in patients when grouped by survivin expression and Ki-67 index

		5-yr Rate, % (95% CI)	10-yr Rate, % (95% CI)	Median Time, months (95% CI)	Median Follow-up, months (Range)
Overall Survival	Total	74 (66–81)	55 (45–64)	139.8 (106.8–156.9)	118.1 (0.9–230.6)
	Ki-67 High / Survivin positive	56 (31–75)	31 (9–56)	76.2 (24.6–188.9)	111.9 (38.7–209.5)
	Ki-67 Low / Survivin positive	63 (48–75)	42 (26–57)	109.7 (52.0–135.9)	121.9 (22.0–174.9)
	Ki-67 Low / Survivin negative	89 (78–95)	71 (55–82)	220.1 (142.7–NR)	122.6 (3.4–230.6)

### Correlation of BIRC5 mRNA expression with RSI in NET patients using genomic dataset

**Figure 4:**
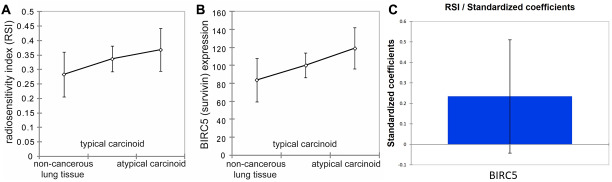
Survivin expression and radio-sensitivity index (RSI) in non-cancerous lung tissues, typical carcinoid, and atypical carcinoid. (**A**) demonstrates that RSI increases in the order of non-cancerous lung tissues, typical carcinoids, and atypical carcinoids. However, Fisher LSD and Tukey HSD show that it is not statistically significantly different between non-cancerous lung tissues and atypical carcinoids, between non-cancerous lung tissues and typical carcinoids, and between typical carcinoids and atypical carcinoids with *p* = 0.118, *p* = 0.235, and *p* = 0.466, respectively. (**B**) represents BIRC5 expression, demonstrating that BIRC5 expression increases in the order of non-cancerous lung tissues, typical carcinoids, and atypical carcinoids. Fisher LSD and Tukey HSD show *p* = 0.039, *p* = 0.243, and *p* = 0.163 between non-cancerous lung tissues and atypical carcinoids, between non-cancerous lung tissues and typical carcinoids, and between typical carcinoids and atypical carcinoids, respectively. Error bars represent standard deviation. (**C**) shows a slight positive correlation between BIRC5 expression and RSI among all tissues R = 0.234, *p* < 0.0001.

We examined the survivin mRNA (BIRC5) expression and RSI in lung NET (typical carcinoid = 31, atypical carcinoid = 11) as well as non-cancerous lung tissue (*n* = 10) using data deposited in Gene Expression Omnibus (GEO) database by Asiedu et al. Within this limited dataset, we found a non-significant trend of increasing BIRC5 mRNA expression with progressive dysplasia in lung NET in the order of non-cancerous lung tissues, typical carcinoid and atypical carcinoid (Figure 4B). Similarly, RSI also increased when going from normal lung tissue to typical carcinoid to atypical carcinoid, although the difference was statistically not significant (Figure 4A). However, when grouped together, correlation analysis of BIRC5 and RSI reveals a Pearson’s coefficient (R) = 0.234 with *p <* 0.0001, suggesting that the expression of BIRC5 and RSI positively correlates with statistical significance (Figure 4C). Correlation was highest in atypical carcinoid group (R = 0.442, *p* = 0.0172). We also found genomic data for 8 pancreatic NET in TCGA and a positive correlation was found between BIRC5 mRNA expression and RSI in pancreatic NET as well (R = 0.824, *p* = 0.012).

### Ionizing radiation enhances BIRC5 gene expression in NCI-H720 human lung carcinoid cells

**Figure 5:**
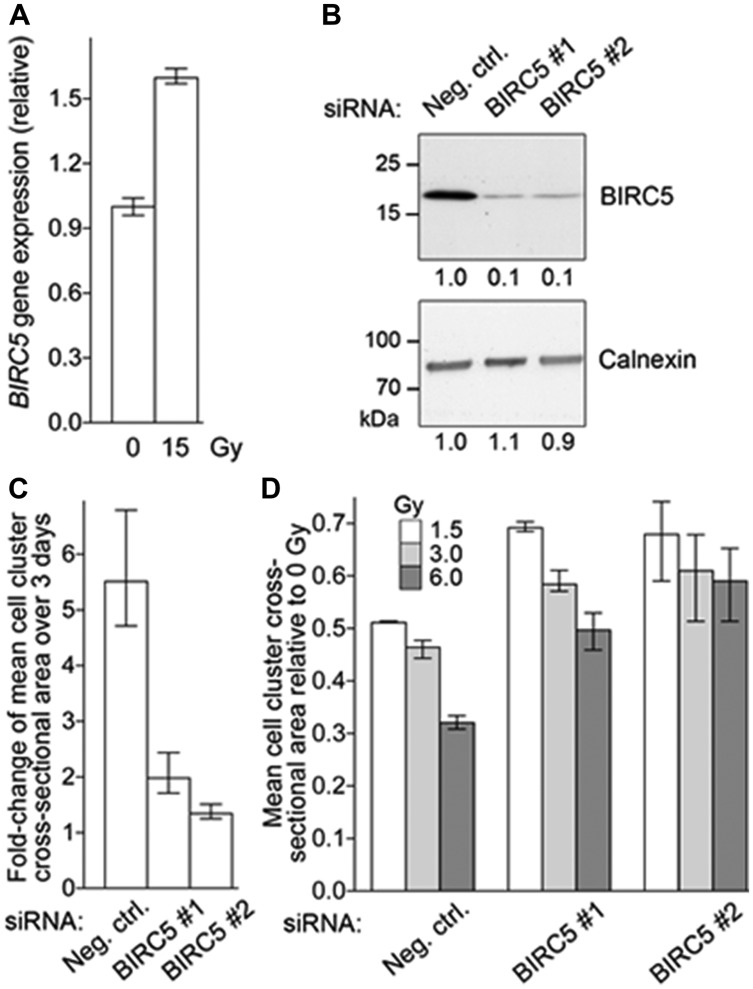
BIRC5 mRNA Expression in NET Cell Line. (**A**). Radiation increases *BIRC5* gene expression of NCI-H720 cells. Cells growing as clusters in suspension in triplicate wells were subjected to one dose of 15 Gy X ray radiation. Control cells (0 Gy) were not subjected to radiation. After 2 days, *BIRC5* gene expression normalized to that of housekeeping ACTB gene was quantified by reverse transcription-PCR. Mean and range (*n* = 3) of relative *BIRC5* expression are depicted. (**B**) siRNA-mediated BIRC5 knock-down in NCI-H720 cells. Single-cell suspensions of NCI-H720 were transfected with a non-specific siRNA (Neg. ctrl.) or with one of two siRNAs against *BIRC5* (*BIRC5*#1 and #2) at a concentration of 8 nM. Whole cell lysates were prepared from the transfectants after 2 days and subjected to immunoblotting to detect BIRC5 and housekeeping calnexin proteins. Different portions of the same blot were used to detect the two proteins. Relative band intensities as measured by image densitometry are listed. (**C**) BIRC5 knock-down reduces NCI-H720 proliferation. Cells were transfected with siRNAs as described for panel B. Average cross-sectional area of cell clusters in cultures of transfectant cells 6 and 9 days after siRNA transfection was determined by quantitative analysis of light microscopy images. Mean and range (*n* = 3) of fold-change in the average cross-sectional area of cell clusters during the 3 days are depicted. (**D**) BIRC5 knock-down does not enhance radiation sensitivity of NCI-H720 cells. Cells were transfected with siRNAs as described for panel B and transfectants were dissociated a day later into single-cell suspensions (0.2 million cells/ml) and immediately treated with a single dose of radiation (1.5, 3, or 6 Gy). Average cross-sectional area of cell clusters in cultures of transfectant cells 6 days after radiation was determined by quantitative analysis of light microscopy images. Mean and range (*n* = 4) of average cell cluster radius relative to non-irradiated (0 Gy) cells are shown.

To examine the effect of radiation on *BIRC5* gene expression, NCI-H720 human lung carcinoid cells were irradiated in triplicate with one 15 Gy dose of ionizing radiation (X rays), and gene expression was measured two days later by RT-PCR. Compared to non-irradiated cells, *BIRC5* expression was 1.6-fold higher in irradiated cells (*t*-test *P* <0.01; Figure 5A).

### Knock-down of BIRC5 reduces proliferation of NCI-H720 cells

Two different siRNAs, each targeting all three transcript variants of human *BIRC5*, could knock-down BIRC5 protein levels in NCI-H720 cells after transfection at a concentration of 8 nM. Compared to a non-specific siRNA, the knock-down efficiency with each of the two siRNAs was >80% (Figure 5B). Proliferation of the transient BIRC5 siRNA transfectants, as assessed from increase in average sizes of cell clusters (cross-sectional area), was significantly reduced, by between 64% to 75% compared to cells transfected with the non-specific siRNA (*t*-test *p* <0.01 for both siRNAs; Figure 5C).

### Knock-down of BIRC5 does not enhance radiation sensitivity of NCI-H720 cells

Because NCI-H720 cells grow in clusters in suspension, we examined their sensitivity to ionizing radiation by monitoring their proliferation through measurement of the average sizes of the cell clusters. Cells were transfected with siRNAs and cell clusters were dissociated a day later. The single-cell suspensions (at 0.2 million cells/ml, and in quadruplicate wells of 24-well tissue culture plates) were subjected to one dose of 1.5, 3, or 6 Gy of ionizing radiation (X rays). Average cell cluster dimension (cross-sectional area) at 6 days after radiation was determined and compared to that determined for cells that were not subjected to radiation. As shown in Figure 5D, 1.5 Gy radiation reduced the average cell cluster dimension was by 49% in the negative control siRNA transfectants, whereas the reductions were only 31%-32% for the two BIRC5 siRNA transfectants. This indicates that BIRC5 knock-down does not enhance radiation sensitivity of NCI-H720 cells. This was also observed for 3 and 6 Gy radiation does (Figure 5D).

## DISCUSSION

There is considerable variation in the clinical course of NET and a need to identify biomarkers that can predict response to available therapies and prognosis. Here, we report the expression of survivin on NET and its correlation with clinical variables and outcomes. Our results show that survivin is associated with aggressive tumor biology as reflected by higher grade and correlation with Ki-67 index, as well as inferior prognosis manifesting as a shorter overall survival. These results are in line with previous studies exploring survivin expression in NET. In a retrospective analysis of 50 patients with well-differentiated gastro-enteropancreatic (GEP) neuroendocrine carcinomas (NEC), nuclear survivin expression by IHC was associated with worse overall survival (OS of 41 vs. 103 months, *p* = 0.001) [15]. However, this study did not include NET originating outside of gastrointestinal tract. Fotouhi at al. further explored the role of survivin in NET and studied its expression in small intestinal NET cell lines using liquid chromatography-mass spectrometry [16]. Their results showed that survivin expression decreased in response to treatment with lanreotide and inhibition of survivin using a small molecule resulted in dramatic reduction in cell proliferation suggesting it has prognostic value. Our study confirms the prognostic role of survivin in all NETs and confirms it is a potential target in NET. The use of a validated assay in current use for other ongoing studies for survivin vaccine is a strength of this study.

Furthermore, we found that lung NET, which are less responsive compared to other NETs to PRRT, have higher survivin expression than GEP NETs. We hypothesized that survivin may play a role in radiation resistance and explored this further. On genomic analysis of a public online database, we found that there is a trend towards higher survivin gene expression (BIRC5) and higher RSI in lung NET with increasing degree of atypia, although it remains statistically not significant. This is likely given the sample size was very small as there is paucity of genomic data for NET. Even with this limited data, there was a positive correlation between survivin expression and RSI meaning that BIRC5 mRNA may play a role in response to radiation therapy. Similar results were found in the 8 patients with pancreatic NET, where radiation sensitivity was higher and is ironically the subgroup of NETs who have the highest response to PRRT. This can have clinical implications in the era of PRRT since the effector mechanism of this therapy is cell damage through radiation. Our experiments with a human NET cell line showed increase in survivin expression in response to radiation which provides more direct evidence to the role of survivin in development of resistance in NET. Although we did not see an increase in radiation sensitivity after knockdown of survivin through siRNA, we noticed that survivin knockdown resulted in cell death in majority of survivin expressing cells. Hence, we are unable to draw any conclusions about the effects of blocking survivin on radiation sensitivity of NET cells *in vitro*.

To date, this is the first analysis of survivin in all NETs including lung NETs and exploration of the correlation between survivin and radiation response. These findings are important due to lack of lung NETs in randomized controlled trials and extrapolation of data from gastroenteropancreatic NETs to lung. Everolimus was approved for metastatic NET of lungs based on results of Radiant-4 trial, which included 90 patients with lung NET and those patients had a hazard ratio of 0.5 (95% CI 0.28 – 0.88) for PFS with everolimus compared to placebo [17]. Somatostatin analogues are used in metastatic lung NET despite lack of randomized studies by extrapolation of data in GEP NET [18, 19]. The recently reported NETTER-1 trial of PRRT in somatostatin receptor positive midgut NET which led to the approval of ^177^Lu-Dotatate in the United States did not include patients with lung NET [20]. Data from an Italian phase II study showed that PRRT is efficacious in lung NETs, with a disease control rate of 80% and PFS of 20 months [21]. Several retrospective studies have demonstrated the antitumor efficacy of PRRT in lung NETs as well [22–24].

The authors acknowledge the shortcoming of a retrospective review, difference in standard of care over the long follow up (samples collected from 1990-2017 with median follow up of 9.3 years). The genomic data did show correlation despite the limited number of cases highlighting the paucity of genomic data in NETs to have the power to make strong conclusions. Further studies in other cell lines and banked lung NET tissues from patients who have has PRRT are needed as cell death was noted in NET cell line after survivin knockdown preventing definitive assessment of the potential of survivin targeting in improving radiation sensitivity.

There has been considerable development in the last decade in targeting survivin through small molecule inhibitors or immunotherapy. One small molecule inhibitors, terameprocol (EM-1421), has been evaluated in early phase studies involving patients with gliomas and advanced leukemias [25, 26]. The immunogenicity of survivin is well established through the detection of survivin-specific cytotoxic T lymphocytes (CTL) and anti-survivin antibodies in serum of some cancer patients [27, 28]. These observations led to attempts at creating a vaccine that can trigger a stronger immune response against survivin. Several vaccine approaches targeting survivin have been evaluated in clinical or pre-clinical studies, including dendritic cell vaccines, DNA vaccines and peptide vaccines, with variable responses [29–33]. More recently, researchers at Roswell Park developed a survivin long peptide-mimic vaccine, SurVaxM, that was shown to generate survivin-specific immunological response through activation of CD8+ CTL as well as CD4+ helper T cells [34]. The safety and immunogenicity of SurVaxM in humans was evaluated by Fenstermaker et al. in a clinical study involving nine patients with survivin positive recurrent malignant gliomas [35]. In this phase I trial, SurVaxM was well-tolerated and majority of patients developed both cellular and humoral immune response to vaccine. Although not designed for survival analysis, the study showed significantly improved median progression-free survival (PFS) and median OS, 17.6 weeks and 86.6 weeks respectively, compared to historical cohorts. A phase II trial evaluating the safety and efficacy of SurVaxM in patients with newly diagnosed survivin positive glioblastoma in adjuvant setting is currently ongoing (NCT02455557). Based on the safety and efficacy signal of this vaccine in malignant gliomas that are survivin positive by IHC and our finding of survivin being a prognostic marker and present in NETs, we find survivin to be a potential target in NET and have begun a pilot trial of SurVaxM in survivin expressing NETs. It’s association with RSI makes it an attractive candidate to be targeted using combination strategies using PRRT upon completion of further preclinical studies testing SurVaxM, survivin antibodies and CAR T-cell approaches to guide optimal therapy and sequencing schedule.

## MATERIALS AND METHODS

### NET cases

All patients were diagnosed and treated at Roswell Park Comprehensive Cancer Center between 1990 and 2017. Inclusion criteria were diagnosis of neuroendocrine neoplasm and availability of tissue specimen for staining by immunohistochemistry. Patients with incomplete records or who did not receive any treatment were excluded. Tissue microarrays (TMAs) were generated representing 167 surgically resected NET specimens and used for immunohistochemistry. These specimens were collected as part of several prospective studies at our institute with consent that allowed exploratory correlative studies. TMAs were stained for survivin and Ki-67. Retrospective chart review was conducted to obtain age, gender, tumor characteristics (diagnosis, morphological grade, site, stage, T and N classification and proliferation marker Ki-67), types of therapy, follow-up and outcomes. Progression-free survival (PFS) was defined as the time period from the 1^st^ line of treatment until detection of recurrence, clinical progression of baseline disease or death. Overall survival (OS) was defined as the period between initial diagnosis and death or last follow up. This study was approved by the local institutional review board.

### Immunohistochemistry

Immunohistochemistry (IHC) staining was performed at the pathology department of Roswell Park Comprehensive Cancer Center. Formalin-fixed paraffin sections were cut at 4 μm, placed on charged slides, and dried at 60°C for one hour. Slides were cooled to room temperature and added to the Dako Omnis autostainer, where they were deparaffinized with Clearify (American Mastertech; catalog #CACLEGA) and rinsed in water. For survivin staining, Flex TRS High (Dako; catalog #GV804) was used for target retrieval for 30 minutes. Slides were incubated with ready to use Survivin antibody (BioSB #BSB2225 clone EP119) for 20 minutes followed by HRP for 20 mins (Dako GV823). DAB (Diaminobenzidine) (Dako; catalog #K3468) was applied for 5 minutes for visualization. Slides were counterstained with Hematoxylin for 10 minutes then placed into water. After removing slides from the Omnis, they were dehydrated, cleared and cover slipped. For TPH staining, Flex TRS Low (Dako; catalog #GV805) was used for target retrieval for 30 minutes. Slides were incubated with Tryptophan Hydroxylase (Sigma #SAB4503029) at 1/50 for 30 minutes followed by HRP for 20 mins (Dako GV823). When available, the data for Ki-67 was retrieved from patient’s chart. For older cases if Ki-67 values were missing, IHC for Ki-67 was performed. Slides were incubated with an anti-Ki 67 antibody (Dako; catalog #M7240) in antibody diluent at room temperature for 60 minutes. The reaction product was revealed using Dako kit 50087. Slides were counterstained with Hematoxylin for 8 minutes and scored per guidelines.

All slides were scanned digitally and then independently evaluated by two board certified pathologists (JQ and AC) who were blinded to patient demographics. Scoring criteria used was the percentage of positive nuclear staining for overall tumor in 5% increments for survivin and classified as present or absent. In case of TPH, intensity of cytoplasmic staining was graded from 0–3 with 0 = no staining, 1 = mild staining, 2 = moderate staining and 3 = strong staining; and patients were grouped as TPH negative (staining ≤1% cells) or TPH positive (staining >1% cells) [36]. Ki-67 staining was evaluated according to the percentage of nuclear staining in the field with the highest percentage of staining, defined after assessing the entire slide. Patients were also classified according to Ki-67 index as low (<3%) or high (≥3%).

### NET genomic database

Expression of mRNA of non-cancerous lung tissue (*n* = 10), typical carcinoid (*n* = 31) and atypical carcinoid (*n* = 11) was downloaded from the data source deposited in the Gene Expression Omnibus (GEO) database with Accession Number GSE 10855. Gene expression data were loaded in the Beadstudio v3 software (Illumina Inc., San Diego, CA), and data were normalized using the cubic spline method [37]. Among two data sets, the normalized data set was used for an analysis of gene expression. Survivin expression (BIRC5) in tissue groups was compared.

Radio-sensitivity index (RSI) developed by researchers at Moffitt Cancer Center is based on the rank-based linear regression of ten genes identified significant for response to radiation therapy [38–40]. Higher RSI’s represent poorer response to radiation therapy. The linear regression of RSI is as follows:

$$\begin{array}{l} \text{RSI }=\;-0.0098009\;\times \text{ AR }+\;0.0128283\;\times \text{ cJun}\\ +\;0.0254552\;\times \text{ STAT1 }-\;0.0017589\;\times \text{ PKC }\\ -\;0.0038171\;\times \text{ RelA}+0.1070213\;\times \text{ cABL}\\ -\;0.0002509\;\times \text{ SUMO}1\;-\;0.0092431\;\times \text{ PAK}2\;\\ -\;0.0204469\;\times \text{ HDAC}-\;0.0441683\;\times \text{ IRF}1 \end{array}$$

### Culture of NCI-H720 lung carcinoid cells

NCI-H720 human atypical lung carcinoid cells, were purchased from American Type Culture Collection (ATCC^®^; Manassas, VA). As recommended by ATCC^®^, the cells were grown in regular tissue culture dishes and flasks at 37° C under 5% CO_2_ and 95% humidity in 1:1 v/v mix of Dulbecco’s modified Eagle’s and Ham’s F12 media (product 10-090-CV, Corning^®^ Corning, NY) supplemented with β-estradiol (10 nM final concentration; product E2758, Sigma^®^, St. Louis, MO), fetal bovine serum (5% v/v; VWR^®^, Radnor, PA), Glutamax™ (1×; product 35050-061, Thermo Scientific^®^, San Diego, CA), hydrocortisone (10 nM; product H0396, Sigma^®^), insulin (5 μg/ml; product 12585-014, Thermo Scientific^®^), sodium selenite (30 nM; product S5261, Sigma^®^), and transferrin (10 μg/ml; product T8158, Sigma^®^). Under these culture conditions, the cells grow in roughly spherical clusters in suspension and proliferate with a doubling time of approximately two days, and Accutase™ (BioLegend^®^, San Diego, CA) was used for cell detachment when splitting cultures.

### Quantification of BIRC5 mRNA of NCI-H720 cells by reverse transcription (RT)-PCR

Total RNA was isolated from cells using an affinity spin-column-based kit (Norgen Biotek^®^, Thorold, Canada). Complementary DNA was generated from RNA in RT reactions with M-MuLV reverse transcriptase and random DNA hexamers, and was used as template in real-time quantitative PCR reactions that were set up with FastStart™ Universal SYBR Green Master PCR mix (Roche^®^, Indianapolis, IN) and run on an LightCycler™ 480 II instrument (Roche^®^). Following primer pairs, that have been used in other studies [41, 42], were used at PCR annealing temperature of 60° C. *ACTB* (β-actin): AGC CTC GCC TTT GCC GA and CTG GTG CCT GGG GCG. *BIRC5* (isoform 1): AGA ACT GGC CCT TCT TGG AGG and CTT TTT ATG TTC CTC TAT GGG GTC. PCR quantification cycle (C_q_) values, which are approximately inversely proportional to log_2_ analyte amplicon concentrations, were determined by LightCycler™ 480 software. C_q_ values of duplicate PCR reactions were averaged and *BIRC5* C_q_ values were normalized by subtracting the C_q_ values for *ACTB*, which was assumed to be a housekeeping gene with stable gene expression.

### Quantification of BIRC5 protein of NCI-H720 cells by western assay

Whole cell protein lysates were prepared with RIPA buffer – 25 mM Tris HCl, 150 mM NaCl, 1% v/v NP-40, 1% w/v sodium deoxycholate, and 0.1% w/v sodium dodecyl sulfate; pH 7.4) with 1× Halt™ protease/phosphatase inhibitor cocktail (Thermo Scientific^®^). Protein lysates (10 μg per lane) were subjected to standard denaturing, reducing polyacrylamide gel (10%) electrophoresis and transferred to a polyvinyl-difluoride membrane. Tris-buffered saline (20 mM Tris HCl and 150 mM NaCl; pH 7.4) with 0.05% v/v Tween-20 and 5% w/v non-fat dry milk was used as buffer to probe the membrane with antibodies against BIRC5 (71G4B7 rabbit monoclonal antibody, product 2808, Cell Signaling Technology^®^, Danvers, MA; used at 1:1000 dilution) or calnexin (rabbit polyclonal antibody, product GTX109669, GeneTex^®^, Irvine, CA; used at 1:5000 dilution). Antibodies bound to their targets on the membrane were detected through chemiluminescence and its detection on radiographic films following the binding of a horseradish peroxidase-conjugated goat antibody against anti-rabbit IgG (product 31460, Thermo Scientific^®^). Intensities of protein bands were determined by densitometry of scanned radiograms with NIH ImageJ software.

### BIRC5 knock-down in NCI-H720 cells using siRNAs

Two Silencer™ Select siRNAs, BIRC5 #1 and #2 (products s1457 and s1458, Thermo Scientific^®^), both of which target all three known BIRC5 transcript variants, were transfected separately into NCI-H720 cells at a final concentration of 8 nM using Lipofectamine RNAiMAX™ reagent (Thermo Scientific^®^). For negative control, Silencer™ Select Negative Control No. 1 siRNA (product 4390843, Thermo Scientific^®^) was transfected. Cells were segregated into single-cell suspension (0.5 million cells/ml) in their culture medium with Accutase™ treatment immediately prior to transfection.

### Treatment of NCI-H720 cells with ionizing radiation

An RX-650 X-ray irradiator (Faxitron^®^, Tucson, AZ) was used to treat cells in 6- or 24-well tissue culture plates with one dose of 1.5, 3, 6, or 15 Gy radiation. Unless noted otherwise, cells were segregated into single-cell suspension (0.2 million cells/ml) in their culture medium with Accutase™ treatment immediately prior to radiation. An EOS 450D digital camera (Canon^®^, Lake Success, NY) and an Axio™ Observer microscope (Zeiss^®^, Maple Grove, MN) with 2.5× objective were used to image the wells under visible light 6 or 9 days after irradiation. Images were analyzed with NIH ImageJ software to quantify 2-dimensional (cross-sectional) areas of cell clusters. Clusters with areas equivalent to <10 cells were ignored.

### Statistical analysis

For statistical analysis of IHC results, comparisons were made using the Mann-Whitney U and Fisher’s exact tests for continuous and categorical variables respectively at α = 0.05. The correlation between survivin and Ki-67 was evaluated using the Spearman correlation coefficient. Survival outcomes were summarized by survivin positivity and Ki-67 using standard Kaplan-Meier methods and the log-rank test. Propensity adjusted analyses were conducted using inverse probability weighted Cox regression models. All analyses were completed in SAS v9.4 (Cary, NC) at a significance level of 0.05.

The results of genomic data base were analyzed by grouping survivin expression (BIRC5) in tissue groups via Analysis of Variance (ANOVA), Fisher Least Significant Difference (LSD) and Tukey Honestly Significant Difference (HSD) with a confidence interval of 95% to assess statistical significance in each pair. The expressivity of RSI and BIRC5 was compared in pairs with non-cancerous lung tissue, typical carcinoid, and atypical carcinoid. ANOVA, Fisher LSD, and Tukey HSD with a confidence interval of 95% were used.

For interpretation of results of cell line experiments, statistical analyses and graph plotting were done with Prism™ software (version 7; GraphPad Software^®^, La Jolla, CA). All t-tests were two-tailed, assumed equal variances, and used 0.05 as the cut-off for deciding significance.

## CONCLUSIONS

Our results indicate that survivin expression in NET is associated with aggressive biology and inferior outcomes. Its expression in pulmonary and pancreatic NET correlates with presence of well-established radiation response genes and may predict resistance to radiation therapy. In human NET cell lines, survivin expression increased after treatment with radiation. Optimizing knock out strategies for survivin in appropriate preclinical models can help to further explore the impact of survivin blockage on radiation sensitivity. Due to the lack of therapies for patients with advanced NET, our work supports exploring survivin as a therapeutic target.
